# Analysis of Clinical Drug-Drug Interaction Data To Predict Magnitudes of Uncharacterized Interactions between Antiretroviral Drugs and Comedications

**DOI:** 10.1128/AAC.00717-18

**Published:** 2018-06-26

**Authors:** Felix Stader, Hannah Kinvig, Manuel Battegay, Saye Khoo, Andrew Owen, Marco Siccardi, Catia Marzolini

**Affiliations:** a Division of Infectious Diseases and Hospital Epidemiology Departments of Medicine and Clinical Research, University Hospital Basel, Basel, Switzerland; b Infectious Disease Modelling Unit, Department of Epidemiology and Public Health, Swiss Tropical and Public Health Institute, Basel, Switzerland; c University of Basel, Basel, Switzerland; d Department of Molecular and Clinical Pharmacology, Institute of Translational Medicine, University of Liverpool, Liverpool, United Kingdom

**Keywords:** drug-drug interaction, antiretroviral drug, CYP3A, HIV infection, comedication

## Abstract

Despite their high potential for drug-drug interactions (DDI), clinical DDI studies of antiretroviral drugs (ARVs) are often lacking, because the full range of potential interactions cannot feasibly or pragmatically be studied, with some high-risk DDI studies also being ethically difficult to undertake. Thus, a robust method to screen and to predict the likelihood of DDIs is required. We developed a method to predict DDIs based on two parameters: the degree of metabolism by specific enzymes, such as CYP3A, and the strength of an inhibitor or inducer. These parameters were derived from existing studies utilizing paradigm substrates, inducers, and inhibitors of CYP3A to assess the predictive performance of this method by verifying predicted magnitudes of changes in drug exposure against clinical DDI studies involving ARVs. The derived parameters were consistent with the FDA classification of sensitive CYP3A substrates and the strength of CYP3A inhibitors and inducers. Characterized DDI magnitudes (*n* = 68) between ARVs and comedications were successfully quantified, meaning 53%, 85%, and 98% of the predictions were within 1.25-fold (0.80 to 1.25), 1.5-fold (0.66 to 1.48), and 2-fold (0.66 to 1.94) of the observed clinical data. In addition, the method identifies CYP3A substrates likely to be highly or, conversely, minimally impacted by CYP3A inhibitors or inducers, thus categorizing the magnitude of DDIs. The developed effective and robust method has the potential to support a more rational identification of dose adjustment to overcome DDIs, being particularly relevant in an HIV setting, given the treatment's complexity, high DDI risk, and limited guidance on the management of DDIs.

## INTRODUCTION

Drug-drug interactions (DDIs) are an increasing burden for patients infected with human immunodeficiency virus (HIV), because DDIs can lead to drug toxicity and treatment failure. Most antiretroviral drugs (ARV) are substrates and inhibitors or inducers of cytochrome P450 (CYP) enzymes, especially the CYP3A family (1), comprised of CYP3A4, CYP3A5, CYP3A7, and CYP3A43 (2). ARVs are considered to be among the therapeutic agents with the highest DDI potential (3). Large surveys suggest around a quarter of HIV patients on treatment are at risk of a clinically significant DDI even with modern antiretroviral therapies (4–6). The risk for DDIs increases significantly with age, multiple morbidities, and polypharmacy (7–9).

It is not feasible, practical, or affordable for every potential DDI to be clinically studied, either during drug development or postlicensing. Some DDIs (e.g., those resulting in increased exposure of narrow therapeutic index drugs) cannot easily or ethically be studied. However, the ability to quantitatively predict the likelihood of any DDI remains important to understand the clinical significance and whether any DDI can be safely managed through dose modification and to prevent unnecessary denial of important therapies to patients. Where data are lacking, empirical expert opinion prevails based on (i) the metabolic pathway of the drugs involved, (ii) *in vitro* drug metabolism data, and (iii) prior knowledge from other DDI studies investigating the effect of paradigm agents on the drug of interest. However, it is challenging to scale these data in any quantitatively useful manner. Furthermore, *in vitro* data as well as clinical DDI trials are often undertaken only for strong, paradigm CYP3A inhibitors (e.g., ketoconazole and itraconazole) and inducers (e.g., rifampin). It is difficult to estimate if and to what extent the dose of the victim drug should be adjusted when administered with a weaker CYP3A inhibitor than ketoconazole or inducer than rifampin. Therefore, a quantitative tool for estimating DDIs involving ARVs could support a more rational dose adjustment to overcome DDIs.

Physiologically based pharmacokinetic models (PBPK) can be used to simulate the magnitude of change in exposure of any given victim drug in a virtual patient population (10), using data obtained from *in vitro* experiments about the susceptibility of the victim and the strength of perpetrator drugs. However, PBPK models are intended to be generally used for predicting DDIs (11) and not to manage daily DDI queries in the clinic.

An alternative method to predict DDIs is to analyze clinical pharmacokinetic data from similar drug combinations with known DDI magnitudes (12, 13). This method can provide a valuable evaluation and prediction of a DDI magnitude, delineating a potential dose adjustment to overcome the DDI effect and ensure a safe and effective treatment of the patient.

The objective of this work was to investigate if DDIs between ARVs and comedications can be quantified from existing clinical pharmacokinetic data of similar drug combinations, providing a rational framework to support necessary dose optimization for HIV-infected patients.

## RESULTS

### Calculation of parameters (fraction of the disposition pathway mediated by CYP3A [DPI_3A_] and CYP3A inhibitor and inducer ratios [InR_3A_/IcR_3A_]) needed for the prediction of DDI magnitudes.

A total of 68 DDIs issued from clinical studies involving ARVs were identified, including 13 involving competitive inhibition (whereby the inhibitor binds to the active site of the enzyme and thereby blocks the binding of the victim drug), 33 involving mechanism-based inhibition (whereby the inhibitor represses the transcription or translation of the metabolizing enzyme, thereby leading to the loss of the enzyme), and 22 involving induction.

The fraction of CYP3A-mediated metabolism for victim drugs (Table 1) and the strength of perpetrators (Table 2) have been calculated for ARVs using published, clinical area under the curve (AUC) ratios of characterized DDIs. Drugs being highly metabolized by CYP3A were triazolam (DPI_3A_, 0.966 [0.898, 1.0]; results are reported as geometric means [confidence intervals]), midazolam (DPI_3A_, 0.940 [0.909, 0.971]), quetiapine (DPI_3A_, 0.845 [0.645, 1.0]), tacrolimus (DPI_3A_, 0.831 [0.531, 1.0]), and maraviroc (DPI_3A_, 0.792 [0.559, 0.998]), whereas CYP3A was only responsible for 30.3% of zolpidem disposition (DPI_3A_, 0.303 [0.065, 0.661]).

**TABLE 1:** Calculated fraction of the disposition pathway mediated by CYP3A (DPI_3A_) of victim drugs sorted by their CYP3A sensitivities^*b*^

Drug	DPI_3A_		Reference(s)^*a*^	Predicted AUC*/AUC with:			
				Ritonavir (inhibitor)		Etravirine (inducer)	
	Single calculation	Monte Carlo simulation		Single calculation	Monte Carlo simulation	Single calculation	Monte Carlo simulation
Simvastatin	0.978	0.979 [0.916, 1.0]	12	45.97	34.54 [5.33, 100.0]	0.34	0.31 [0.12, 0.44]
Triazolam	0.959	0.966 [0.898, 1.0]	30, 31	24.39	24.97 [4.91, 100.0]	0.35	0.31 [0.12, 0.44]
Midazolam	0.940	0.940 [0.909, 0.971]	32–35	16.67	12.36 [5.40, 20.64]	0.35	0.32 [0.13, 0.45]
Quetiapine	0.896	0.845 [0.645, 1.0]	37	9.66	7.97 [2.42, 62.46]	0.36	0.34 [0.14, 0.47]
Tacrolimus	0.875	0.831 [0.531, 1.0]	36	8.03	10.20 [1.97, 100.0]	0.37	0.34 [0.14, 0.48]
Maraviroc	0.828	0.792 [0.559, 0.998]	38	5.80	5.85 [1.98, 31.26]	0.38	0.35 [0.14, 0.49]
Saquinavir	0.638	0.580 [0.258, 0.849]	39	2.76	2.88 [1.31, 6.04]	0.44	0.42 [0.18, 0.57]
Darunavir	0.602	0.543 [0.215, 0.817]	40	2.51	2.62 [1.26, 5.19]	0.46	0.43 [0.18, 0.59]
Macitentan	0.585	0.524 [0.200, 0.810]	41	2.41	2.55 [1.24, 4.98]	0.47	0.43 [0.19, 0.59]
Amlodipine	0.468	0.464 [0.117, 1.0]	16	1.88	2.75 [1.12, 30.13]	0.52	0.46 [0.19, 0.64]
Etravirine	0.340	0.336 [0.077, 0.701]	42	1.52	1.79 [1.08, 3.18]	0.60	0.52 [0.24, 0.71]
Rilpivirine	0.324	0.334 [0.079, 0.656]	43	1.48	1.73 [1.08, 2.84]	0.61	0.53 [0.24, 0.71]
Zolpidem	0.268	0.303 [0.065, 0.661]	44, 45	1.37	1.66 [1.06, 2.79]	0.66	0.55 [0.25, 0.73]
Ritonavir	0.156	0.231 [0.038, 0.552]	46	1.19	1.46 [1.04, 2.19]	0.77	0.60 [0.29, 0.78]

a References reporting observed AUC ratios used to calculate DPI_3A_ according to equation 3. If more than one study reported the AUC ratio for the same drug combination, the weighted mean was used to derive DPI_3A_.

b Single calculations not considering variability and Monte Carlo simulations (*n* = 10,000) were performed to predict DDI magnitudes with the inhibitor ritonavir and the inducer etravirine. Results of the Monte Carlo simulations are presented as geometric means [95% confidence intervals].

**TABLE 2:** Calculated inhibitor (InR_3A_) and inducer (IcR_3A_) ratios sorted by their strengths^*b*^

Drug	InR_3A_/IcR_3A_		Reference(s)^*a*^	Predicted AUC*/AUC with:			
				Maraviroc		Rilpivirine	
	Single calculation	Monte Carlo simulations		Single calculation	Monte Carlo simulations	Single calculation	Monte Carlo simulations
Ritonavir	1.0	0.963 [0.873, 1.0]	18–22	5.80	5.85 [1.98, 7.63]	1.48	1.73 [1.08, 2.06]
Ketoconazole	0.981	0.943 [0.823, 1.0]	47	5.32	4.61 [1.93, 6.25]	1.47	1.64 [1.08, 2.00]
Cobicistat	0.967	0.936 [0.810, 1.0]	9, 34, 48–51	5.00	5.35 [1.91, 7.04]	1.46	1.71 [1.08, 2.00]
Voriconazole	0.951	0.916 [0.768, 1.0]	52	4.69	4.67 [1.78, 6.26]	1.45	1.67 [1.08, 1.98]
Itraconazole	0.920	0.877 [0.691, 0.986]	53	4.19	4.14 [1.72, 5.46]	1.43	1.62 [1.07, 1.92]
Clarithromycin	0.912	0.870 [0.680, 0.982]	54	4.08	3.98 [1.66, 5.24]	1.42	1.61 [1.07, 1.89]
Saquinavir	0.858	0.803 [0.557, 0.946]	55	3.45	3.32 [1.53, 4.34]	1.39	1.54 [1.07, 1.80]
Posaconazole	0.852	0.797 [0.551, 0.944]	50, 56	3.39	3.28 [1.53, 4.29]	1.38	1.54 [1.06, 1.79]
Diltiazem	0.780	0.715 [0.427, 0.898]	17	2.82	2.70 [1.36, 3.46]	1.34	1.46 [1.05, 1.68]
Fluconazole	0.768	0.700 [0.403, 0.893]	33	2.74	2.62 [1.34, 3.38]	1.33	1.45 [1.05, 1.66]
Cimetidine	0.213	0.256 [0.049, 0.570]	57–59	1.21	1.38 [1.04, 1.57]	1.07	1.15 [1.01, 1.23]
Efavirenz	0.566	0.539 [0.282, 0.850]	60	0.48	0.43 [0.18, 0.61]	0.70	0.60 [0.29, 0.79]
Etravirine	0.662	0.644 [0.433, 0.882]	42	0.38	0.35 [0.14, 0.49]	0.61	0.53 [0.24, 0.71]
Rifampin	0.946	0.906 [0.846, 0.967]	39	0.06	0.10 [0.04, 0.20]	0.15	0.20 [0.07, 0.29]

a References reporting observed AUC ratios used to calculate InR_3A_ and IcR_3A_ according to equations 4 and 5. If more than one study reported the AUC ratio for the same drug combination, the weighted mean was used to derive InR_3A_/IcR_3A_.

b Single calculations not considering variability and Monte Carlo simulations (*n* = 10,000) were performed to predict DDI magnitudes with the victim drugs maraviroc, a sensitive CYP3A substrate, and rilpivirine, a moderate CYP3A substrate. Results of the Monte Carlo simulations are presented as geometric means [95% confidence intervals].

Ritonavir (InR_3A_, 0.963 [0.873, 1.0]), cobicistat (InR_3A_, 0.936 [0.810, 1.0]), and ketoconazole (InR_3A_, 0.943 [0.823, 1.0]) were estimated to be potent CYP3A inhibitors, whereas cimetidine was the weakest inhibitor of CYP3A in this study (InR_3A_, 0.256 [0.049, 0.570]). The moderate inducers efavirenz and etravirine had IcR_3A_ values of 0.539 [0.282, 0.850] and 0.644 [0.433, 0.882], whereas the strong inducer rifampin had an IcR_3A_ of 0.906 [0.846, 0.967].

### Verification of the method.

**FIG 1:**
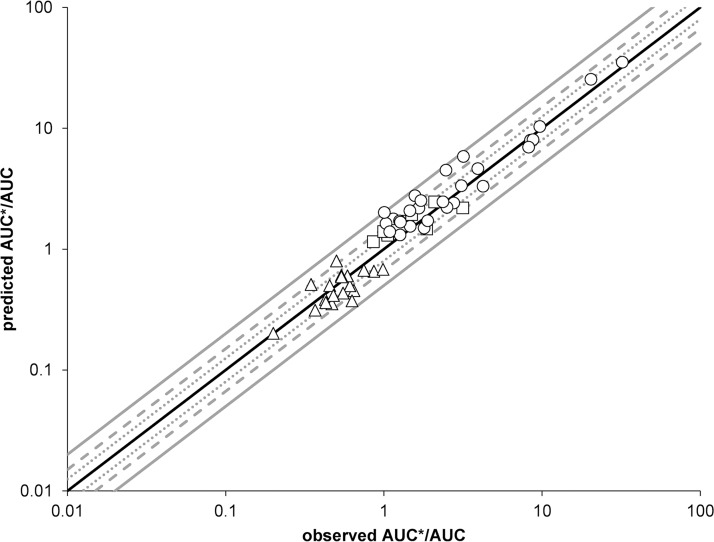
Predicted versus observed AUC ratios for competitive inhibition (open squares), mechanism-based inhibition (open circles), and induction (open triangles). The solid black line is the line of identity, the dotted lines represent the 80% and 125% margins, the dashed lines represent the 66% and 150% margins, and the solid gray lines represent the 50% and 200% margins. The interaction between saquinavir and cimetidine was calculated by using the InR_3A_ of the stronger inhibitor saquinavir rather than that of cimetidine, as explained in Discussion.

Characterized DDIs involving ARVs have been calculated by using the derived DPI and InR/IcR values to verify the method and investigate its potential to predict uncharacterized DDIs (Fig. 1). More than half of the predictions (53%) were within 1.25-fold (ratio predicted/observed, 0.80 to 1.25), 85% of the predicted values were within 1.5-fold (ratio predicted/observed: 0.66 to 1.48), and 98% of the predictions were within 2-fold (ratio predicted/observed, 0.66 to 1.98) of the observed clinical data for all investigated DDI mechanisms. Overall, predictions were equally performant for ARVs acting as perpetrators (ritonavir, cobicistat, darunavir, saquinavir, etravirine, and efavirenz) or as victim drugs (maraviroc and rilpivirine).

### Impact and interplay of DPI_3A_ and InR_3A_/IcR_3A_.

To analyze the impact of DPI_3A_, the interaction magnitude with the potent inhibitor ritonavir (InR_3A_, 0.963 [0.873, 1,0]) and the moderate inducer etravirine (IcR_3A_, 0.644 [0.433, 0.882]) have been calculated (Table 1). Drugs being highly metabolized by CYP3A, like triazolam and maraviroc, showed a >5-fold increase in exposure when given with ritonavir and roughly a 65% decrease in the AUC when administered with etravirine. Monte Carlo simulations allowed us to investigate the variability of predictions, which can be remarkably high for some interactions (e.g., tacrolimus when administered with ritonavir or etravirine).

The impact of InR_3A_ and IcR_3A_ was investigated by calculating the DDI potential with the victim drugs maraviroc, for which CYP3A is responsible for 79.2% of the disposition (DPI_3A_, 0.792 [0.559, 0.998]), and rilpivirine, which is less metabolized by CYP3A (DPI_3A_, 0.334 [0.079, 0.656]) (Table 2). Strong CYP3A inhibitors like ritonavir and cobicistat led to a 5-fold increase in the AUC of maraviroc and to a 1.5-fold increase of rilpivirine exposure, showing also the impact of DPI_3A_. This holds true for inducers, because CYP3A substrates like maraviroc and rilpivirine were more impacted by rifampin than efavirenz and etravirine. The predicted variability was greater for maraviroc, being highly metabolized by CYP3A, particularly for inhibitors and inducers impacting CYP3A strongly, than for rilpivirine.

**FIG 2:**
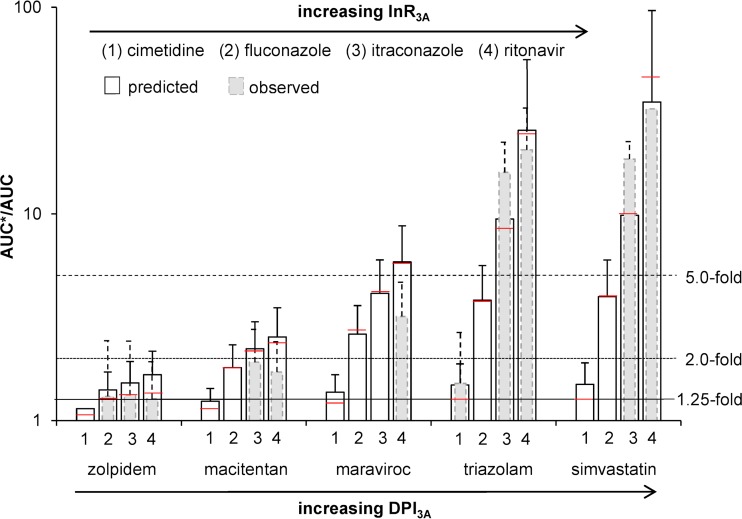
Comparison of predicted (white bars) and observed (gray bars) AUC ratios plus standard deviations for the five victim drugs zolpidem (DPI_3A_, 0.303 [0.065, 0.661]), macitentan (DPI_3A_, 0.524 [0.200, 0.810]), maraviroc (DPI_3A_, 0.792 [0.559, 0.998]), triazolam (DPI_3A_, 0.966 [0.898, 1.0]), and simvastatin (DPI_3A_, 0.979 [0.916, 1.0]), administered together with the four CYP3A inhibitors cimetidine (InR_3A_, 0.256 [0.049, 0.570]), fluconazole (InR_3A_, 0.700 [0.403, 0.893]), itraconazole (InR_3A_, 0.877 [0.691, 0.986]), and ritonavir (InR_3A_, 0.963 [0.873, 1.0]). The red line represents the calculated value without using Monte Carlo predictions. The solid line, the dotted line, and the dashed line represent the 1.25-fold, 2.0-fold, and 5.0-fold increases in the AUC ratio according to the FDA classification of DDI magnitudes (29).

To investigate the interplay between the role CYP3A plays in the metabolism of drugs (represented by DPI_3A_) and the inhibitor strength (represented by InR_3A_), the DDI magnitude between five victim drugs with increasing DPI_3A_ (zolpidem < macitentan < maraviroc < triazolam < simvastatin) and inhibitors with increasing InR_3A_ (cimetidine < fluconazole < itraconazole < ritonavir) have been investigated and compared to observed clinical data, if available (Fig. 2). The strong inhibitors ritonavir and itraconazole led to a >5-fold increase of exposure for victim drugs being highly metabolized by CYP3A, whereas drugs that were not as sensitive to CYP3A, like zolpidem (DPI_3A_, 0.303 [0.065, 0.661]), resulted in a weak AUC ratio increase of less than 2-fold. Moderate CYP3A inhibitors like fluconazole gave rise to a moderate DDI magnitude (2- to 5-fold), and CYP3A inhibitors with low InR_3A_ values like cimetidine resulted in low DDI magnitudes (<1.25) when administered with a drug being highly metabolized by CYP3A (e.g., maraviroc, triazolam, and simvastatin). Therefore, the magnitude of a DDI depends on the sensitivity of a substrate toward a specific metabolic pathway, represented by DPI, and the strength of an inhibitor or inducer, represented by InR and IcR.

### Autoinhibition and autoinduction of ARVs.

**FIG 3:**
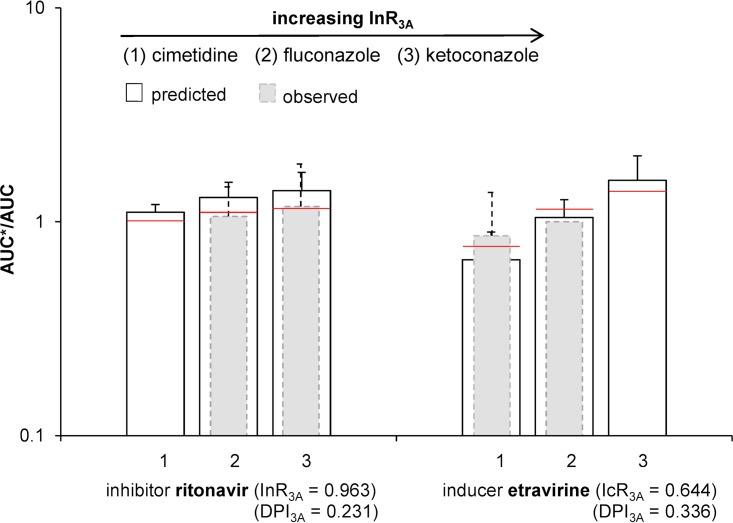
Comparison of the predicted (white bars) and observed (gray bars) AUC ratio plus standard deviations for the two victim drugs with intrinsic inhibitory (ritonavir) and inducing (etravirine) properties administered together with the three CYP3A inhibitors cimetidine (InR_3A_, 0.256 [0.049, 0.570]), fluconazole (InR_3A_, 0.700 [0.403, 0.893]), and ketoconazole (InR_3A_, 0.943 [0.823, 1.0]). The red line represents the calculated value without using Monte Carlo predictions.

It should be highlighted that some ARVs, for instance, ritonavir and etravirine, can inhibit or induce their own metabolism (autoinhibition/autoinduction), which is important to consider when quantifying DDIs involving those drugs. The inhibitor ritonavir (InR_3A_, 0.963 [0.873, 1.0]; DPI_3A_, 0.231 [0.038, 0.552]) and the inducer etravirine (IcR_3A_, 0.644 [0.433, 0.882]; DPI_3A_, 0.336 [0.077, 0.701]) were used as victim drugs, and the effects of the CYP3A inhibitors cimetidine (weak inhibitor), fluconazole (moderate inhibitor), and ketoconazole (strong inhibitor) on ritonavir and etravirine exposures were investigated (Fig. 3). Even with strong inhibitors like ketoconazole, there was only a minor effect on ritonavir and etravirine exposure, which is in accordance with observed clinical data.

### Prediction of uncharacterized DDIs between tyrosine kinase inhibitors and ARVs.

Tyrosine kinase inhibitors being mainly metabolized by CYP3A (ibrutinib and dasatinib, with DPI_3A_ values of 0.971 [0.916, 1.0] and 0.779 [0.524, 1.0]), given with the potent CYP3A inhibitor ritonavir, led to strong DDIs, with AUC ratios of >5-fold. Considering variability estimated by Monte Carlo simulations, all predicted AUC ratios of ibrutinib given with ritonavir were above 5-fold. Moderate DDIs were predicted for tyrosine kinase inhibitors being intermediately metabolized by CYP3A (lapatinib, nilotinib, and gefitinib, with DPI_3A_ values of 0.696 [0.403, 0.936], 0.646 [0.333, 0.895], and 0.534 [0.197, 0.884], respectively), although some AUC ratios were above 5-fold and therefore categorized as strong DDIs. Tyrosine kinase inhibitors for which CYP3A is a minor pathway (pazopanib, sunitinib, and imatinib, with DPI_3A_ values of 0.383 [0.106, 0.700], 0.336 [0.079, 0.661], and 0.301 [0.064, 0.627]) showed weak DDIs when administered with ritonavir, although considering variability, some predicted AUC ratios had a magnitude greater than 2-fold, classifying them as moderate DDIs. Sorafenib is almost not metabolized by CYP3A (DPI_3A_ of 0.054 [0.006, 0.391]) and showed no DDI with ritonavir.

**FIG 4:**
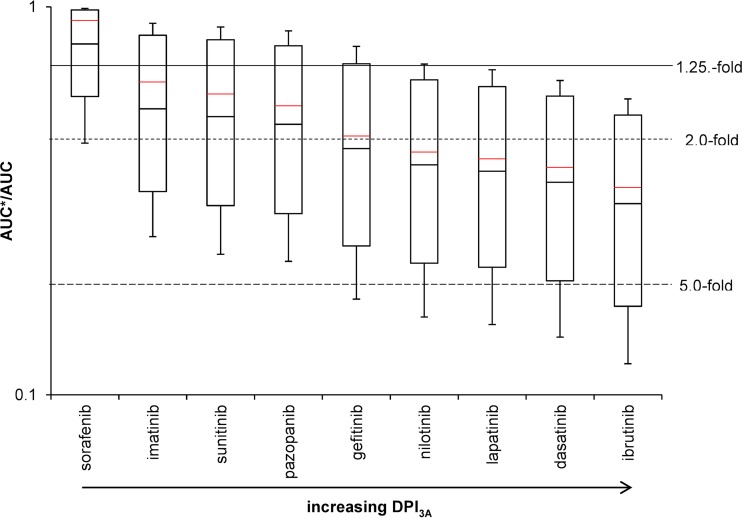
DDI magnitudes between tyrosine kinase inhibitors with increasing DPI_3A_ and the moderate CYP3A inducer etravirine predicted by running 10,000 Monte Carlo simulations. The solid red line shows calculated values without using Monte Carlo predictions. The solid line, the dotted line, and the dashed line represent the 1.25-fold, 2.0-fold, and 5.0-fold increase in the AUC ratio according to the FDA classification of DDI magnitudes (29).

Results for the tyrosine kinase inhibitors were similar when administered with the moderate inducer etravirine (Fig. 4). Tyrosine kinase inhibitors being highly or intermediately metabolized by CYP3A showed moderate DDIs when given with etravirine, with some predicted AUC ratios being above 5-fold. Administration of etravirine with imatinib, sunitinib, and pazopanib led to weak DDIs. The combination of sorafenib and etravirine showed no DDI potential.

## DISCUSSION

The management of DDIs involving ARVs is often problematic in clinical practice, as guidance on dosing recommendations is only provided for a limited number of evaluated drug combinations. We present a robust and reproducible method to predict uncharacterized DDIs between ARVs and comedications. Of interest, the method discriminates between CYP3A substrates likely to be significantly, or else marginally, impacted by CYP3A inhibitors and inducers, and therefore categorizes DDIs according to their magnitude. The method has the potential to support a more rational identification of dose adjustment to overcome DDIs with the perspective to help guide DDI management in the future. In addition, this approach can help to minimize and rationalize future clinical studies, providing a valuable characterization of the interaction potential. The integration with modeling techniques such as PBPK will additionally allow us to have a comprehensive prediction of DDI magnitude as well as identify drug combinations that might require more detailed laboratory and clinical investigations.

The method predicts DDIs based on the degree of metabolism by a specific enzyme, such as CYP3A (represented by DPI), and the strength of an inhibitor or inducer (represented by InR and IcR). These parameters can be estimated from existing clinical pharmacokinetic studies and are used, in turn, to estimate uncharacterized DDIs involving ARVs. The derived parameters, DPI_3A_ and InR_3A_/IcR_3A_, were consistent with the FDA classification (14). CYP3A-sensitive substrates such as triazolam, midazolam, and tacrolimus had DPI_3A_ values close to 1. Conversely, drugs being partly metabolized by CYP3A, like zolpidem, had lower DPI_3A_ values and were shown to be less sensitive to CYP3A inhibitors or inducers, because metabolism can still occur through unaffected CYPs. This example highlights how multiple metabolic pathways can modulate the magnitude of DDIs. Furthermore, ritonavir, cobicistat, and ketoconazole were predicted to be strong inhibitors, whereas fluconazole was found to be a moderate inhibitor. The strength of inducers was also correctly predicted, with rifampin characterized as a strong inducer and etravirine or efavirenz as moderate inducers. Thus, the parameters necessary for the method can be reliably derived from existing clinical DDI studies.

The derived parameters were used subsequently to predict DDIs and were compared to existing clinical DDI studies to assess the predictive performance of the method. All predicted AUC ratios were within 2-fold of the observed data, apart from saquinavir and cimetidine. Additionally, 53% and 85% of all predictions fell within 1.25- and 1.5-fold (Fig. 1) of the observed data. The reason for the underprediction of the saquinavir-cimetidine interaction could be that cimetidine is a weaker inhibitor of CYP3A than saquinavir. It is likely that CYP3A is not fully inhibited by cimetidine, thus the inhibitory effect of the protease inhibitor saquinavir adds up to the total inhibition effect, while strong CYP3A inhibitors like ketoconazole inhibit the enzyme completely, thus an additive effect by protease inhibitors would not be detectable (10). Therefore, in the situation of two combined drugs both having the potential to inhibit CYP3A, the use of the inhibitor ratio (InR) of the stronger inhibitor among the two drugs is recommended to obtain a more accurate prediction. In this case, the InR_3A_ of saquinavir should be used to calculate the DDI magnitude between saquinavir and cimetidine, leading to a predicted AUC ratio of 2.23, which is in accordance with the observed one of 2.48 (ratio predicted/observed, 0.90).

**FIG 5:**
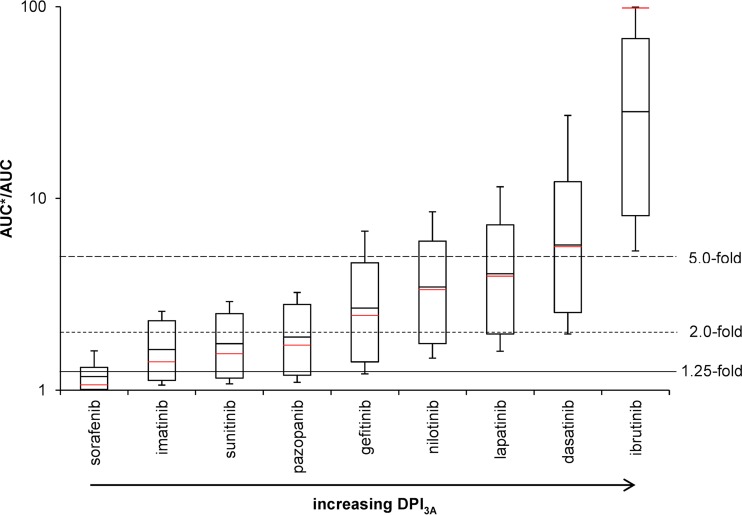
DDI magnitudes between tyrosine kinase inhibitors with increasing DPI_3A_ and the potent CYP3A inhibitor ritonavir predicted by running 10,000 Monte Carlo simulations. The solid red line shows the calculated value without using Monte Carlo predictions. The solid line, the dotted line, and the dashed line represent the 1.25-fold, 2.0-fold, and 5.0-fold increases in the AUC ratio according to the FDA classification of DDI magnitudes (29).

Of importance, this method allows us to categorize the magnitude of DDIs. DDIs of large magnitude are expected for drugs being highly metabolized by a given enzyme in the presence of strong or moderate inhibitors and inducers of this enzyme (Fig. 2). Therefore, in this situation, depending on the dose-exposure relationship of the victim drug, dose adjustment and/or close clinical monitoring might be needed unless the drug combination leads to deleterious effects, for instance, the combination of dasatinib or ibrutinib and ritonavir, which consequently should be avoided (Fig. 5) (15). Conversely, drugs metabolized by multiple enzymes are expected to be weakly impacted by strong inhibitors or inducers and not affected by moderate and weak perpetrators. Therefore, no dose adjustment would be needed *a priori* unless the drug has a narrow therapeutic index, like tyrosine kinase inhibitors (15). Furthermore, our results illustrate that drugs with strong or at least moderate inhibitory or inducing properties on their own metabolism (e.g., ritonavir or etravirine) are minimally impacted by other inhibitors or inducers, as their own effect on CYPs counteracts the effect of other drugs (Fig. 3).

The strengths of this method are that it can potentially be applied to any given CYP enzyme and for any given DDI, and that results shown for tyrosine kinase inhibitors can be translated to other drug classes. One clinical example would be an HIV-infected patient receiving ritonavir who suffers from hypertension, which requires treatment with amlodipine, a calcium channel inhibitor. Amlodipine is metabolized by CYP3A, and therefore an interaction would be expected with ritonavir. To get an idea about the DDI magnitude, the presented approach is used. The package insert usually contains information about known DDIs and can therefore be used to estimate DPI_3A_ of the chosen drug. The package insert of amlodipine contains the information that diltiazem, a strong CYP3A inhibitor (14), increases amlodipine exposure by 60%, and other strong CYP3A inhibitors, like ketoconazole and ritonavir, may increase amlodipine concentration to a greater extent without further details on the potential DDI magnitude and guidance on how to adjust the amlodipine dose to prevent hypotension and edemas (16). By deriving the inhibitory strength of diltiazem (InR_3A_, 0.715 [0.427,0.898] [Monte Carlo simulations] or 0.802 [single calculation]) using an existing midazolam/diltiazem DDI study (17), the fraction of CYP3A-mediated metabolism of amlodipine can be calculated to be 0.464 [0.117, 1.0] (Monte Carlo simulations) or 0.468 (single calculation). The inhibitory strength of ritonavir was 0.963 [0.873, 1.0] (Monte Carlo simulations) or 1 (single calculation) was derived from midazolam/ritonavir DDI studies (18–22). Both the fraction of amlodipine metabolism mediated by CYP3A and the inhibitory strength of ritonavir were used to calculate the magnitude of the DDI (AUC ratio) using equation 1. The parameters derived from Monte Carlo simulations or single calculation provided similar DDI predictions. A 90% increase in amlodipine exposure is expected, suggesting a 50% dose reduction would be required when amlodipine is given with ritonavir, which is in agreement with findings from a published DDI study (23). It should be noted that the decision to adjust the dose should not be based only on the pharmacokinetic change but should also take into account the possibility to monitor the DDI effect, drug-response relationship, and the therapeutic index to safely manage DDIs.

Some limitations should be acknowledged. It is worth mentioning that perpetrators are never completely selective and therefore affect more than one pathway. Thus, the method cannot distinguish between effects attributed exclusively to the specific CYP enzyme of interest or if the DDI results from a combined effect from other CYPs, UGTs, and/or drug transporters. In addition, the method cannot separate effects from different locations (e.g., intestinal versus hepatic). However, the multiple combined effects of several enzymes and drug transporters and the contribution of different sites to a DDI are accounted for in the prediction, because clinical data of similar DDIs with similar pathways are used. DDIs of drug pairs can be quantified, but in clinical reality subjects may receive concomitantly several drugs, which may interact mutually. Furthermore, the method does not account for genetic background, age, and comorbidities (e.g., liver cirrhosis), which all can impact the magnitude of DDIs. To answer these questions, more mechanistic PBPK models are necessary, which are highly established in the field of DDI predictions (24, 25). The method was verified using the common 2-fold limit, which might be considered too permissive for drugs with a narrow therapeutic index (26). However, the potential underprediction of a DDI would occur only if the inhibitor/inducer of interest alters a pathway other than that of the paradigm perpetrator used to derive the necessary parameters (DPI and InR/IcR). It is therefore recommended that DPI and InR/IcR be derived using several perpetrators for the prediction of uncharacterized DDIs involving drugs with a narrow therapeutic index and that results are compared to exclude this possibility.

In conclusion, the developed effective and robust method can minimize and rationalize future clinical studies, providing an effective prediction of DDI magnitude. Additionally, it has the potential to support a more rational identification of dose adjustment to overcome DDIs, being particularly relevant in an HIV setting, given the treatment's complexity, high DDI risk, and limited guidance on the management of DDIs. In the future, the method could be extended to other drug classes and prediction of multiple DDIs.

## MATERIALS AND METHODS

This study extends the method to estimate DDIs from clinical data to ARVs (12, 13). The common metric to quantify a DDI is the ratio of AUC of a victim drug in the presence of a perpetrator (AUC*) to the AUC of the victim drug in the absence of the perpetrator. The AUC ratio can be expressed in the following way for inhibitors (equation 1) and inducers (equation 2):

(1) $$\frac{{\text{AUC}}^{*}}{\text{AUC}}=\frac{1}{1-\text{DP}{\text{I}}_{x}\times \text{In}{\text{R}}_{x}}$$

(2) $$\frac{\text{AU}{\text{C}}^{*}}{\text{AUC}}=\frac{1}{\frac{\text{DP}{\text{I}}_{x}}{1-\text{Ic}{\text{R}}_{x}}+(1-\text{DP}{\text{I}}_{x})}$$

where DPI is the fraction of the disposition pathway mediated by a specific enzyme, *x*, such as CYP3A, and InR and IcR are the inhibitor and inducer ratio, respectively. CYP3A was chosen because it is a major contributor to the metabolism of drugs in clinical use and is therefore often involved in DDIs. A detailed description of the method used and the deviation of the equation can be found in the supplemental material.

DPI represents the importance of a metabolic pathway of interest. A DPI_3A_ of 1 means that 100% of the drug is metabolized by CYP3A, hence no other enzyme or transporter is involved in the disposition of the drug, while a DPI_3A_ of 0 means that CYP3A is not involved in the metabolism at all.

InR/IcR reflects the potential of a perpetrator to inhibit or induce the metabolic pathway of interest. An InR_3A_/IcR_3A_ of 1 indicates a high inhibition/induction potential, while a low InR_3A_/IcR_3A_ value translates to a weak inhibition/induction potential.

### Data source for clinical studies to verify the method.

A structured literature search was performed using the MEDLINE database to screen for DDI studies to develop and verify the method. Key words were “drug-drug-interaction,” “effect,” and the victim and perpetrator drugs of interest. Chosen victim drugs should be highly, intermediately, and weakly metabolized by CYP3A, and chosen perpetrators should inhibit or induce CYP3A strongly, moderately, or weakly. Data were included if subjects were healthy volunteers or HIV-infected patients but did not have any severe disease affecting metabolizing enzymes (i.e., liver cirrhosis). At least 80% of the subjects of one study were white, because different ethnicities can have an impact on CYP abundance, and drug administration was oral. If more than one study reported an AUC ratio for the same victim-perpetrator drug combination, the weighted mean was calculated.

### Calculation of parameters (DPI and InR/IcR) used for the prediction of DDI magnitudes.

With a given AUC ratio determined in a clinical study, DPI and InR/IcR of victim and perpetrator drugs can be estimated. Therefore, equations 1 and 2 are rearranged to calculate DPI (equation 3), InR (equation 4), and IcR (equation 5).

(3) $$\text{DP}{\text{I}}_{x}=\frac{1-\frac{\text{AUC}}{\text{AU}{\text{C}}^{*}}}{\text{In}{\text{R}}_{x}}$$

(4) $$\text{In}{\text{R}}_{x}=\frac{1-\frac{\text{AUC}}{\text{AU}{\text{C}}^{*}}}{\text{DP}{\text{I}}_{x}}$$

(5) $$\text{Ic}{\text{R}}_{x}=\frac{1-\frac{\text{AUC}}{\text{AU}{\text{C}}^{*}}}{1-\frac{\text{AUC}}{\text{AU}{\text{C}}^{*}}-\text{DP}{\text{I}}_{x}}$$

**FIG 6:**
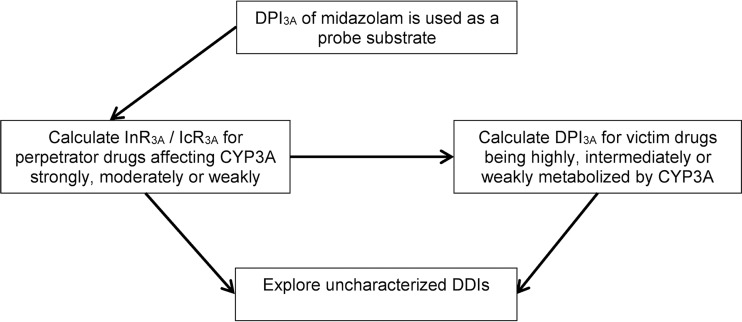
Workflow of the study. DPI_3A_, fraction of disposition pathway mediated by CYP3A; InR_3A_, inhibitor ratio; IcR_3A_, inducer ratio.

To start the method, a model drug with a known DPI_3A_ is needed (Fig. 6). We used midazolam as a probe substrate, which is highly metabolized by CYP3A4/3A5 and, to a lesser extent, UGT1A4 (27). In the first step, InR_3A_ of strong, moderate, and weak inhibitors and IcR_3A_ of strong, moderate, and weak inducers were calculated from DDI studies with midazolam. In the second step, DPI_3A_ was calculated for drugs being highly, intermediately, or weakly metabolized by CYP3A. In the third step, characterized DDI magnitudes were calculated using the derived DPI_3A_ and InR_3A_/IcR_3A_ values in equation 1 or 2, and the predicted results were compared to observed clinical data. Judgement if a prediction was successful was assessed by the 1.25-, 1.5-, and 2-fold method as used by the FDA (28). In the last step, uncharacterized DDI magnitudes were predicted for tyrosine kinase inhibitors being weakly, intermediately, or highly metabolized by CYP3A (sorafenib, imatinib, sunitinib, pazopanib, gefitinib, nilotinib, lapatinib, dasatinib, and ibrutinib), ritonavir as a potent CYP3A inhibitor, and etravirine as a moderate CYP3A inducer. Predicted DDI magnitudes were classified into no DDIs (AUC ratio of 0.8 to 1.25), weak DDIs (AUC ratio of 1.25 to 2.0 for inhibition and 0.5 to 0.8 for induction), moderate DDIs (AUC ratio of 2.0 to 5.0 for inhibition and 0.2 to 0.5 for induction), and strong DDIs (AUC ratio above 5.0 for inhibitors and below 0.2 for inducers) according to the FDA (29).

To capture the observed variability in clinical DDI studies, Monte Carlo simulations (*n* = 10,000) were performed to calculate the equation parameters (DPI_3A_ and InR_3A_/IcR_3A_) and AUC ratios of characterized DDIs to verify the method as well as uncharacterized DDIs between tyrosine kinase inhibitors and ARVs. Results are reported as the geometric means [95% confidence intervals].

## Supplementary Material

Supplemental material

## References

[1] Warnke D, Barreto J, Temesgen Z 2007 Antiretroviral drugs. J Clin Pharmacol 47:1570–1579. doi:10.1177/0091270007308034. 18048575

[2] de Wildt SN, Kearns GL, Leeder JS, van den Anker JN 1999 Cytochrome P450 3A. Clin Pharmacokinet 37:485–505. doi:10.2165/00003088-199937060-00004. 10628899

[3] Barry M, Mulcahy F, Merry C, Gibbons S, Back D 1999 Pharmacokinetics and potential interactions amongst antiretroviral agents used to treat patients with HIV infection. Clin Pharmacokinet 36:289–304. doi:10.2165/00003088-199936040-00004. 10320951

[4] Marzolini C, Elzi L, Gibbons S, Weber R, Fux C, Furrer H, Chave J-P, Cavassini M, Bernasconi E, Calmy A, Vernazza P, Khoo S, Ledergerber B, Back D, Battegay M 2010 Prevalence of comedications and effect of potential drug-drug interactions in the Swiss HIV Cohort Study. Antiviral Ther 15:413. doi:10.3851/IMP1540. 20516560

[5] Miller CD, El-Kholi R, Faragon JJ, Lodise TP 2007 Prevalence and risk factors for clinically significant drug interactions with antiretroviral therapy. Pharmacotherapy 27:1379–1386. doi:10.1592/phco.27.10.1379. 17896893

[6] Evans-Jones JG, Cottle LE, Back DJ, Gibbons S, Beeching NJ, Carey PB, Khoo SH 2010 Recognition of risk for clinically significant drug interactions among HIV-infected patients receiving antiretroviral therapy. Clin Infect Dis 50:1419–1421. doi:10.1086/652149. 20380564

[7] Greene M, Steinman MA, McNicholl IR, Valcour V 2014 Polypharmacy, drug–drug interactions, and potentially inappropriate medications in older adults with human immunodeficiency virus infection. J Am Geriatrics Soc 62:447–453. doi:10.1111/jgs.12695. PMC4043391 24576251

[8] Darque A, Enel P, Petit N, Ravaux I, Retornaz F 2012 Drug interactions in elderly individuals with the human immunodeficiency virus. J Am Geriatrics Soc 60:382–384. doi:10.1111/j.1532-5415.2011.03795.x. 22332689

[9] Tsunoda SM, Velez RL, Moltke LL, Greenblatt DJ 1999 Differentiation of intestinal and hepatic cytochrome P450 3A activity with use of midazolam as an in vivo probe: effect of ketoconazole. Clin Pharmacol Ther 66:461–471. doi:10.1016/S0009-9236(99)70009-3. 10579473

[10] Rostami-Hodjegan A, Tucker G 2004 “In silico” simulations to assess the “in vivo” consequences of “in vitro” metabolic drug–drug interactions. Drug Discov Today Technol 1:441–448. doi:10.1016/j.ddtec.2004.10.002. 24981625

[11] Jamei M 2016 Recent advances in development and application of physiologically-based pharmacokinetic (PBPK) models: a transition from academic curiosity to regulatory acceptance. Curr Pharmacol Rep 2:161–169. doi:10.1007/s40495-016-0059-9. 27226953 PMC4856711

[12] Ohno Y, Hisaka A, Suzuki H 2007 General framework for the quantitative prediction of CYP3A4-mediated oral drug interactions based on the AUC increase by coadministration of standard drugs. Clin Pharmacokinet 46:681–696. doi:10.2165/00003088-200746080-00005. 17655375

[13] Tod M, Goutelle S, Clavel-Grabit F, Nicolas G, Charpiat B 2011 Quantitative prediction of cytochrome P450 (CYP) 2D6-mediated drug interactions. Clin Pharmacokinet 50:519–530. doi:10.2165/11592620-000000000-00000. 21740075

[14] U.S. Food and Drug Administration. 2016 Drug development and drug interactions: table of substrates, inhibitors and inducers. U.S. Food and Drug Administration, Washington, DC https://www.fda.gov/drugs/developmentapprovalprocess/developmentresources/druginteractionslabeling/ucm093664.htm Accessed 9 April 2017.

[15] Teo YL, Ho HK, Chan A 2015 Metabolism-related pharmacokinetic drug-drug interactions with tyrosine kinase inhibitors: current understanding, challenges and recommendations. Br J Clin Pharmacol 79:241–253. doi:10.1111/bcp.12496. 25125025 PMC4309630

[16] Pfizer Laboratories. 2011 Package insert of Norvasc (amlodipine). Pfizer Laboratories, New York, NY https://www.accessdata.fda.gov/drugsatfda_docs/label/2011/019787s047lbl.pdf Accessed 9 December 2017.

[17] Backman JT, Olkkola KT, Aranko K, Himberg J-J, Neuvonen PJ 1994 Dose of midazolam should be reduced during diltiazem and verapamil treatments. Br J Clin Pharmacol 37:221–225. doi:10.1111/j.1365-2125.1994.tb04266.x. 8198928 PMC1364750

[18] Greenblatt DJ, Peters DE, Oleson LE, Harmatz JS, MacNab MW, Berkowitz N, Zinny MA, Court MH 2009 Inhibition of oral midazolam clearance by boosting doses of ritonavir, and by 4, 4-dimethyl-benziso-(2H)-selenazine (ALT-2074), an experimental catalytic mimic of glutathione oxidase. Br J Clin Pharmacol 68:920–927. doi:10.1111/j.1365-2125.2009.03545.x. 20002087 PMC2810804

[19] Yeh RF, Gaver VE, Patterson KB, Rezk NL, Baxter-Meheux F, Blake MJ, Eron JJ Jr, Klein CE, Rublein JC, Kashuba AD 2006 Lopinavir/ritonavir induces the hepatic activity of cytochrome P450 enzymes CYP2C9, CYP2C19, and CYP1A2 but inhibits the hepatic and intestinal activity of CYP3A as measured by a phenotyping drug cocktail in healthy volunteers. J Acquired Immune Defic Syndr 42:52–60. 16639344 10.1097/01.qai.0000219774.20174.64

[20] Schmitt C, Hofmann C, Riek M, Zwanziger E, Patel A 2009 Effect of saquinavir-ritonavir on cytochrome P450 3A4 activity in healthy volunteers using midazolam as a probe. Pharmacotherapy 29:1175–1181. doi:10.1592/phco.29.10.1175. 19792991

[21] Kirby BJ, Collier AC, Kharasch ED, Whittington D, Thummel KE, Unadkat JD 2011 Complex drug interactions of HIV protease inhibitors 1: inactivation, induction and inhibition of cytochrome P450 3A by ritonavir or nelfinavir. Drug Metab Disposition 39:1070–1078. doi:10.1124/dmd.110.037523. PMC3100903 21406602

[22] Knox TA, Oleson L, von Moltke LL, Kaufman RC, Wanke CA, Greenblatt DJ 2008 Ritonavir greatly impairs CYP3A activity in HIV infection with chronic viral hepatitis. J Acquired Immune Defic Syndr 49:358–368. doi:10.1097/QAI.0b013e31818c7efe. 19186349

[23] Glesby MJ, Aberg JA, Kendall MA, Fichtenbaum CJ, Hafner R, Hall S, Grosskopf N, Zolopa AR, Gerber JG 2005 Pharmacokinetic interactions between indinavir plus ritonavir and calcium channel blockers. Clin Pharmacol Ther 78:143–153. doi:10.1016/j.clpt.2005.04.005. 16084849

[24] Rowland-Yeo K, Jamei M, Yang J, Tucker GT, Rostami-Hodjegan A 2010 Physiologically based mechanistic modelling to predict complex drug–drug interactions involving simultaneous competitive and time-dependent enzyme inhibition by parent compound and its metabolite in both liver and gut–the effect of diltiazem on the time-course of exposure to triazolam. Eur J Pharm Sci 39:298–309. doi:10.1016/j.ejps.2009.12.002. 20025966

[25] Rose R, Neuhoff S, Abduljalil K, Chetty M, Rostami-Hodjegan A, Jamei M 2014 Application of a physiologically based pharmacokinetic model to predict OATP1B1-related variability in pharmacodynamics of rosuvastatin. Pharmacometrics Syst Pharmacol 3:1–9. 10.1038/psp.2014.24 PMC4120018 25006781

[26] Guest EJ, Aarons L, Houston JB, Rostami-Hodjegan A, Galetin A 2011 Critique of the two-fold measure of prediction success for ratios: application for the assessment of drug-drug interactions. Drug Metab Disposition 39:170–173. doi:10.1124/dmd.110.036103. 21036951

[27] Galetin A, Ito K, Hallifax D, Houston JB 2005 CYP3A4 substrate selection and substitution in the prediction of potential drug-drug interactions. J Pharmacol Exp Ther 314:180–190. doi:10.1124/jpet.104.082826. 15784650

[28] Wagner C, Pan Y, Hsu V, Grillo JA, Zhang L, Reynolds KS, Sinha V, Zhao P 2015 Predicting the effect of cytochrome P450 inhibitors on substrate drugs: analysis of physiologically based pharmacokinetic modeling submissions to the US Food and Drug Administration. Clin Pharmacokinet 54:117–127. doi:10.1007/s40262-014-0188-4. 25260695

[29] U.S. Food and Drug Administration. 2017 Clinical drug interaction studies–study design, data analysis, and clinical implications. Guidance for industry. U.S. Food and Drug Administration, Washington, DC https://www.fda.gov/downloads/drugs/guidances/ucm292362.pdf Accessed 22 March 2018.

[30] Greenblatt DJ, Wright CE, Moltke LL, Harmatz JS, Ehrenberg BL, Harrel LM, Corbett K, Counihan M, Tobias S, Shader RI 1998 Ketoconazole inhibition of triazolam and alprazolam clearance: differential kinetic and dynamic consequences. Clin Pharmacol Ther 64:237–247. doi:10.1016/S0009-9236(98)90172-2. 9757147

[31] Varhe A, Olkkola KT, Neuvonen PJ 1994 Oral triazolam is potentially hazardous to patients receiving systemic antimycotics ketoconazole or itraconazole. Clin Pharmacol Ther 56:601–607. doi:10.1038/clpt.1994.184. 7995001

[32] Ahonen J, Olkkola KT, Neuvonen PJ 1995 Effect of itraconazole and terbinafine on the pharmacokinetics and pharmacodynamics of midazolam in healthy volunteers. Br J Clin Pharmacol 40:270–272. 8527290 PMC1365108

[33] Olkkola KT, Ahonen J, Neuvonen PJ 1996 The effect of the systemic antimycotics, itraconazole and fluconazole, on the pharmacokinetics and pharmacodynamics of intravenous and oral midazolam. Anesthesia Analgesia 82:511–516. 8623953 10.1097/00000539-199603000-00015

[34] Olkkola KT, Backman JT, Neuvonen PJ 1994 Midazolam should be avoided in patients receiving the systemic antimycotics ketoconazole or itraconazole. Clin Pharmacol Ther 55:481–485. doi:10.1038/clpt.1994.60. 8181191

[35] Backman JT, Kivistö KT, Olkkola KT, Neuvonen PJ 1998 The area under the plasma concentration–time curve for oral midazolam is 400-fold larger during treatment with itraconazole than with rifampicin. Eur J Clin Pharmacol 54:53–58. doi:10.1007/s002280050420. 9591931

[36] Sansone-Parsons A, Krishna G, Martinho M, Kantesaria B, Gelone S, Mant TG 2007 Effect of oral posaconazole on the pharmacokinetics of cyclosporine and tacrolimus. Pharmacotherapy 27:825–834. doi:10.1592/phco.27.6.825. 17542765

[37] Grimm SW, Richtand NM, Winter HR, Stams KR, Reele SB 2006 Effects of cytochrome P450 3A modulators ketoconazole and carbamazepine on quetiapine pharmacokinetics. Br J Clin Pharmacol 61:58–69. doi:10.1111/j.1365-2125.2005.02507.x. 16390352 PMC1884989

[38] Abel S, Russell D, Taylor-Worth RJ, Ridgway CE, Muirhead GJ 2008 Effects of CYP3A4 inhibitors on the pharmacokinetics of maraviroc in healthy volunteers. Br J Clin Pharmacol 65:27–37. doi:10.1111/j.1365-2125.2008.03133.x. 18333863 PMC2311406

[39] Grub S, Bryson H, Goggin T, Lüdin E, Jorga K 2001 The interaction of saquinavir (soft gelatin capsule) with ketoconazole, erythromycin and rifampicin: comparison of the effect in healthy volunteers and in HIV-infected patients. Eur J Clin Pharmacol 57:115–121. doi:10.1007/s002280100277. 11417442

[40] Sekar VJ, Lefebvre E, De Pauw M, Vangeneugden T, Hoetelmans RM 2008 Pharmacokinetics of darunavir/ritonavir and ketoconazole following co-administration in HIV–healthy volunteers. Br J Clin Pharmacol 66:215–221. doi:10.1111/j.1365-2125.2008.03191.x. 18460033 PMC2492919

[41] Atsmon J, Dingemanse J, Shaikevich D, Volokhov I, Sidharta PN 2013 Investigation of the effects of ketoconazole on the pharmacokinetics of macitentan, a novel dual endothelin receptor antagonist, in healthy subjects. Clin Pharmacokinet 52:685–692. doi:10.1007/s40262-013-0063-8. 23568224

[42] Schöller-Gyüre M, Kakuda TN, Raoof A, De Smedt G, Hoetelmans RM 2009 Clinical pharmacokinetics and pharmacodynamics of etravirine. Clin Pharmacokinet 48:561–574. doi:10.2165/10895940-000000000-00000. 19725591

[43] Crauwels H, Van Heeswijk R, Stevens M, Buelens A, Vanveggel S, Boven K, Hoetelmans R 2012 Clinical perspective on drug-drug interactions with the non-nucleoside reverse transcriptase inhibitor rilpivirine. AIDS Rev 15:87–101. 23681436

[44] Greenblatt DJ, Moltke LL, Harmatz JS, Mertzanis P, Graf JA, Durol ALB, Counihan M, Roth-Schechter B, Shader RI 1998 Kinetic and dynamic interaction study of zolpidem with ketoconazole, itraconazole, and fluconazole. Clin Pharmacol Ther 64:661–671. doi:10.1016/S0009-9236(98)90057-1. 9871431

[45] Luurila H, Kivistö KT, Neuvonen PJ 1998 Effect of itraconazole on the pharmacokinetics and pharmacodynamics of zolpidem. Eur J Clin Pharmacol 54:163–166. doi:10.1007/s002280050439. 9626922

[46] Bertz R, Wong C, Carothers L, Lauva I, Dennis S, Valdes J 1998 Evaluation of the pharmacokinetics of multiple dose ritonavir and ketoconazole in combination. Clin Pharmacol Ther 63:230.

[47] Sohlenius-Sternbeck A-K, Meyerson G, Hagbjörk A-L, Juric S, Terelius Y 2018 A strategy for early-risk predictions of clinical drug–drug interactions involving the GastroPlus DDI module for time-dependent CYP inhibitors. Xenobiotica 48:348–356. doi:10.1080/00498254.2017.1323136. 28443803

[48] Lam Y, Alfaro CL, Ereshefsky L, Miller M 2003 Pharmacokinetic and pharmacodynamic interactions of oral midazolam with ketoconazole, fluoxetine, fluvoxamine, and nefazodone. J Clin Pharmacol 43:1274–1282. doi:10.1177/0091270003259216. 14551182

[49] Eap CB, Buclin T, Cucchia G, Zullino D, Hustert E, Bleiber G, Golay KP, Aubert AC, Baumann P, Telenti A 2004 Oral administration of a low dose of midazolam (75 μg) as an in vivo probe for CYP3A activity. Eur J Clin Pharmacol 60:237–246. doi:10.1007/s00228-004-0762-z. 15114429

[50] Krishna G, Moton A, Ma L, Savant I, Martinho M, Seiberling M, McLeod J 2009 Effects of oral posaconazole on the pharmacokinetic properties of oral and intravenous midazolam: a phase I, randomized, open-label, crossover study in healthy volunteers. Clin Ther 31:286–298. doi:10.1016/j.clinthera.2009.02.022. 19302901

[51] Chung E, Nafziger AN, Kazierad DJ, Bertino JS 2006 Comparison of midazolam and simvastatin as cytochrome P450 3A probes. Clin Pharmacol Ther 79:350–361. doi:10.1016/j.clpt.2005.11.016. 16580903

[52] Saari TI, Laine K, Leino K, Valtonen M, Neuvonen PJ, Olkkola KT 2006 Effect of voriconazole on the pharmacokinetics and pharmacodynamics of intravenous and oral midazolam. Clin Pharmacol Ther 79:362–370. doi:10.1016/j.clpt.2005.12.305. 16580904

[53] Neuvonen PJ, Kantola T, Kivistö KT 1998 Simvastatin but not pravastatin is very susceptible to interaction with the CYP3A4 inhibitor itraconazole. Clin Pharmacol Ther 63:332–341. doi:10.1016/S0009-9236(98)90165-5. 9542477

[54] Gorski JC, Jones DR, Haehner-Daniels BD, Hamman MA, O'Mara EM, Hall SD 1998 The contribution of intestinal and hepatic CYP3A to the interaction between midazolam and clarithromycin. Clin Pharmacol Ther 64:133–143. doi:10.1016/S0009-9236(98)90146-1. 9728893

[55] Palkama VJ, Ahonen J, Neuvonen PJ, Olkkola KT 1999 Effect of saquinavir on the pharmacokinetics and pharmacodynamics of oral and intravenous midazolam. Clin Pharmacol Ther 66:33–39. doi:10.1016/S0009-9236(99)70051-2. 10430107

[56] Krishna G, Ma L, Prasad P, Moton A, Martinho M, O'Mara E 2012 Effect of posaconazole on the pharmacokinetics of simvastatin and midazolam in healthy volunteers. Expert Opin Drug Metab Toxicol 8:1–10. doi:10.1517/17425255.2012.639360. 22176629

[57] Greenblatt DJ, Locniskar A, Scavone JM, Blyden GT, Ochs HR, Harmatz JS, Shader RI 1986 Absence of interaction of cimetidine and ranitidine with intravenous and oral midazolam. Anesthesia Analgesia 65:176–180. 2935051

[58] Klotz U, Arvela P, Rosenkranz B 1985 Effect of single doses of cimetidine and ranitidine on the steady-state plasma levels of midazolam. Clin Pharmacol Ther 38:652–655. doi:10.1038/clpt.1985.240. 2933205

[59] Fee J, Collier P, Howard P, Dundee J 1987 Cimetidine and ranitidine increase midazolam bioavailability. Clin Pharmacol Ther 41:80–84. doi:10.1038/clpt.1987.13. 3802710

[60] Gerber JG, Rosenkranz SL, Fichtenbaum CJ, Vega JM, Yang A, Alston BL, Brobst SW, Segal Y, Aberg JA 2005 Effect of efavirenz on the pharmacokinetics of simvastatin, atorvastatin, and pravastatin: results of AIDS Clinical Trials Group 5108 Study. J Acquir Immune Defic Syndr 39:307–312. doi:10.1097/01.qai.0000167156.44980.33. 15980690

