# Supplementary material for: Analysis of Clinical Drug-Drug Interaction Data To Predict Magnitudes of Uncharacterized Interactions between Antiretroviral Drugs and Comedications
Source: Antimicrob Agents Chemother. 2018 Jun 26;62(7):e00717-18. doi: 10.1128/AAC.00717-18 (PMC6021627; doi:10.1128/AAC.00717-18)
Supplement: Supplemental material [file supp_62_7_e00717-18__index.html]

Supplemental material 

# Analysis of Clinical Drug-Drug Interaction Data To Predict Magnitudes of Uncharacterized Interactions between Antiretroviral Drugs and Comedications

## Supplemental material

- Supplemental file 1 -

  Supplemental Text S1

  PDF, 42K
