# Supplementary material for: Analysis of Clinical Drug-Drug Interaction Data To Predict Magnitudes of Uncharacterized Interactions between Antiretroviral Drugs and Comedications
Source: Antimicrob Agents Chemother. 2018 Jun 26;62(7):e00717-18. doi: 10.1128/AAC.00717-18 (PMC6021627; doi:10.1128/AAC.00717-18)
Supplement: Supplemental material [file AAC.00717-18_zac007187245s1.pdf]

Supplement

A full deviation of the used equation is provided here. The oral clearance of a drug A is defined as followed.

$$CL_{oral} = \frac{CL_{tot}}{F} = \frac{CL_{hep}+CL_{ren}+CL_{add}}{fa \times F_G \times F_H} \tag{1}$$

where CL_{tot} = total clearance, F = bioavailability, CL_{hep} = total hepatic clearance, CL_{ren} = renal clearance, CL_{add} = additional clearance not via liver or kidney, f_a = fraction absorbed, F_G = fraction escaping gut metabolism and F_H = fraction escaping first pass metabolism.

The following assumptions hold true.

1) The main route of metabolism is the liver. Renal or other pathways are assumed to be negligible.

$$CL_{ren} = CL_{add} = 0 \tag{2}$$

2) The orally administered drug is fully absorbed.

$$fa = 1 \tag{3}$$

3) The well-stirred liver model holds true.

$$CL_{hep} = \frac{Q_H \times CL_{int} \times fu_B}{Q_H+CL_{int} \times fu_B} \tag{4}$$

$$F_H = \frac{Q_H}{Q_H+CL_{int} \times fu_B} \tag{5}$$

where Q_H = liver blood flow, CL_{int} = total intrinsic hepatic clearance, fu_B = fraction unbound in blood.

4) Unbound concentration in the intracellular space of the liver and the plasma are similar

5) The metabolic pathway follows Michaelis-Menten kinetics.

6) The intracellular, unbound substrate concentration is below K_M and therefore clearance of the substrate is independent of the dose.

Considering the assumptions and equations 2, 3, 4 and 5, equation 1 changes as followed:

$$CL_{oral} = \frac{\frac{Q_H \times CL_{int} \times fu_B}{Q_H+CL_{int} \times fu_B}+0+0}{1 \times F_G * \frac{Q_H}{Q_H+CL_{int} \times fu_B}} = \frac{CL_{int} \times fu_B}{F_G} \tag{6}$$

In the presence of a perpetrator, oral clearance of drug A changes as followed:

$$CL_{oral}^{*} = \frac{CL_{int}^{*} \times fu_B}{F_G^{*}} \quad (7)$$

Where the superscript * stands for in the presence of the perpetrator.

The common metric to assess an interaction of drug A and the perpetrator is the AUC ratio.

$$\frac{AUC^{*}}{AUC} = \frac{\frac{Dose}{CL_{oral}^{*}}}{\frac{Dose}{CL_{oral}}} = \frac{CL_{oral}}{CL_{oral}^{*}} = \frac{\frac{CL_{int} \times fu_B}{F_G}}{\frac{CL_{int}^{*} \times fu_B}{F_G^{*}}} = \frac{CL_{int} \times F_G^{*}}{CL_{int}^{*} \times F_G} \quad (8)$$

It is assumed that gut metabolism is not affected by the interaction.

$$\frac{F_G^{*}}{F_G} = 1 \quad (9)$$

The total intrinsic hepatic clearance depends on enzymes, which metabolize drug A. In this work, we are interested in CYP3A, but the method is general and can potentially be used for all CYP enzymes.

$$CL_{int} = fm_{3A} \times CL_{int} + (1 - fm_{3A}) \times CL_{int} \quad (10)$$

Where *fm* = fraction metabolized by a certain enzyme.

Now we assume to have a perpetrator only affecting the CYP3A pathway.

$$CL_{int}^{*} = \frac{fm_{3A} \times CL_{int}}{FR_{3A}} + (1 - fm_{3A)} \times CL_{int} \quad (11)$$

Where *FR* is the fold reduction by the perpetrator. FR depends on the type of interaction, which can be competitive inhibition (equation 12), mechanism-based inhibition (equation 13) or induction (equation 14).

$$FR_{3A} = 1 + \frac{[I]}{K_i} \quad (12)$$

$$FR_{3A} = 1 + \frac{k_{inact} * [I]}{k_{deg} * (K_{app} + [I])} \quad (13)$$

$$FR_{3A} = \frac{1}{1 + \frac{IndMax * [I]}{[I] + IC_{50}}} \quad (14)$$

Where *[I]* = inhibitor concentration at steady state, K_i = inhibition constant, k_{deg} = enzyme degradation rate, k_{inact} = inactivation rate of an enzyme for mechanism-based inhibition, K_{app} = apparent enzyme inhibition constant for mechanism-based inhibition (concentration of the inhibitor associated with half maximum inactivation rate), Ind_{max} = maximum fold of induction and IC_{50} = half maximum inhibitory concentration.

Considering equation 9, 10 and 11, the AUC ratio can be written as followed.

$$\frac{AUC^*}{AUC} = \frac{CL_{int}}{CL_{int}^*} = \frac{CL_{int}}{\frac{fm_{3A} \times CL_{int}}{FR_{3A}} + (1 - fm_{3A}) \times CL_{int}} = \frac{CL_{int}}{CL_{int} \times \left(\frac{fm_{3A}}{FR_{3A}} + (1 - fm_{3A})\right)} = \frac{1}{\frac{fm_{3A}}{FR_{3A}} + 1 - fm_{3A}} \quad (15)$$

The inhibitor ratio (*InR*) was defined by Hisaka et al. [1] for competitive inhibition, but can be used for mechanism-based inhibition as well.

$$InR_{3A} = 1 - \frac{1}{1 + \frac{[I]}{K_i}} = 1 - \frac{1}{FR_{3A}} \quad (16)$$

The inducer ratio (*IcR*) can be defined in a similar way as the inhibitor ratio, but the reciprocal needs to be used.

$$IcR_{3A} = 1 - FR_{3A} \quad (17)$$

FR in equation 15 can now be replaced by InR (equation 16) and IcR (equation 17). Because the method cannot distinguish between different inhibited enzymes and transporters, fm_{3A} needs to be replaced by the broadly defined fraction of disposition pathway altered by the perpetrator (DPI_{3A}).

For CYP3A inhibitors, the AUC-ratio can be calculated according to equation 18 and for inducers according to equation 19.

$$\frac{AUC^*}{AUC} = \frac{1}{\frac{DPI_{3A}}{FR_{3A}} + (1 - DPI_{3A})} = \frac{1}{1 - DPI_{3A} * \left(1 - \frac{1}{FR_{3A}}\right)} = \frac{1}{1 - DPI_{3A} * InR_{3A}} \quad (18)$$

$$\frac{AUC^*}{AUC} = \frac{1}{\frac{DPI_{3A}}{1 - IcR_{3A}} + (1 - DPI_{3A})} \quad (19)$$

An example for the method is a patient infected with HIV with high blood pressure that needs treatment with amlodipine (see discussion for more information). In this case, the DPI_{3A} of amlodipine is calculated from a known DDI study between amlodipine and diltiazem described in the package insert of

amlodipine. In this example, single predictions instead of Monte-Carlo simulations are performed which is more realistic in supporting clinical DDI queries. As shown in the manuscript, both approaches give similar results.

$$DPI_{3A}(amlodipine) = \frac{1 - \frac{AUC}{AUC^*}}{InR_{3A}(diltiazem)} = \frac{1 - \frac{1}{1.6}}{0.802} = 0.468 \quad (20)$$

By knowing the inhibitory strength of ritonavir and the fraction of amlodipine metabolism by CYP3A4, the DDI magnitude between amlodipine and ritonavir can be calculated:

$$\frac{AUC^*}{AUC} = \frac{1}{1 - DPI_{3A}(amlodipine) \times InR_{3A}(ritonavir)} = \frac{1}{1 - 0.468 \times 1} = 1.9 \quad (21)$$

1. Hisaka A, Yoshiyuki O, Yamamoto T, & Suzuki H. 2010. Theoretical considerations on quantitative prediction of drug-drug interactions. Drug Metabolism and Pharmacokinetics 25(1): 48-61.
